# The Influence of Anion Shape on the Electrical Double Layer Microstructure and Capacitance of Ionic Liquids-Based Supercapacitors by Molecular Simulations

**DOI:** 10.3390/molecules22020241

**Published:** 2017-02-16

**Authors:** Ming Chen, Song Li, Guang Feng

**Affiliations:** 1 State Key Laboratory of Coal Combustion, School of Energy and Power Engineering, Huazhong University of Science and Technology, Wuhan 430074, China; mchen@hust.edu.cn (M.C.); gfeng@hust.edu.cn (G.F.); 2 Nano Interface Centre for Energy, School of Energy and Power Engineering, Huazhong University of Science and Technology, Wuhan 430074, China; 3 Shenzhen Research Institute of Huazhong University of Science and Technology, Shenzhen 518057, China

**Keywords:** supercapacitor model, ion shape, carbon electrode, interfacial structure, capacitance

## Abstract

Room-temperature ionic liquids (RTILs) are an emerging class of electrolytes for supercapacitors. In this work, we investigate the effects of different supercapacitor models and anion shape on the electrical double layers (EDLs) of two different RTILs: 1-ethyl-3-methylimidazolium bis(trifluoromethanesulfonyl)imide ([Emim][Tf_2_N]) and 1-ethyl-3-methylimidazolium 2-(cyano)pyrrolide ([Emim][CNPyr]) by molecular dynamics (MD) simulation. The EDL microstructure is represented by number densities of cations and anions, and the potential drop near neutral and charged electrodes reveal that the supercapacitor model with a single electrode has the same EDL structure as the model with two opposite electrodes. Nevertheless, the employment of the one-electrode model without tuning the bulk density of RTILs is more time-saving in contrast to the two-electrode one. With the one-electrode model, our simulation demonstrated that the shapes of anions significantly imposed effects on the microstructure of EDLs. The EDL differential capacitance vs. potential (C-V) curves of [Emim][CNPyr] electrolyte exhibit higher differential capacitance at positive potentials. The modeling study provides microscopic insight into the EDLs structure of RTILs with different anion shapes.

## 1. Introduction

The limited availability of fossil fuels and the urgent demand for environmentally friendly and sustainable energy resources bring about an intense need for efficient energy storage devices, leading to widespread research efforts into electrochemical capacitors, especially into supercapacitors, in the past decade [1,2,3]. In contrast to batteries, electrical double layer capacitors (EDLCs), one kind of supercapacitor, store charge via adsorbing counter-ions to balance the charge on the electrodes, and exhibit higher power density, faster charge-discharge rate and longer cycle life [4]. With high surface area, electrical conductivity and wide accessibility, carbon-based materials, for instance, carbide-derived carbons [5,6], carbon fibers [7,8,9], carbon nanotubes (CNTs) [9,10,11], carbons onions [12], and graphene-based materials [13,14], have been widely exploited as the supercapacitor electrodes. Regarding electrolytes, compared with the conventional aqueous and organic electrolytes, room-temperature ionic liquids (RTILs) [15,16,17] are considered as promising candidates for electrolytes used in supercapacitors due to their unique characteristics, including a widened electrochemical window, good ionic conductivity, non-flammability, and low vapor pressure [17,18,19,20,21,22].

The structure of the electrical double layers (EDLs) at the interfaces of RTILs and the electrode has been intensively studied in experiment, theory and modeling. Ohsaka and co-workers studied the EDL capacitance near carbon and gold electrodes with imidazolium tetrafluoroborate RTILs by the impedance technique and found that the capacitance-electrical potential (C-V) curves were parabolic-like shaped at the glassy carbon electrode [23]. Lockett and Sedev et al. studied the influences of cation size and temperature on the differential capacitance of imidazolium-based ionic liquids and reported the camel-like C-V curve from impedance measurements [24]. Fedorov and Kornyshev proposed a new formula to predict that the C-V curve of RTILs consisting of similar sized cations and anions should be bell- or camel-shaped, which depends on the so-called lattice-saturation parameter, γ, the ratio of the bulk density of the ions in the liquid to the maximal possible density in the EDL [25]. Our previous study has investigated the effects of different anion sizes and electrode curvatures on the EDL structure of supercapacitors by molecular dynamics (MD) simulation and found that the size of anions could affect EDL capacitance due to the variation in cation-anion interactions for RTILs with varying cation/anion size ratios [26]. It was also revealed that the cation/anion size ratio imposes great impacts on the shape of C-V curves: the smaller the cation/anion size ratio is, the higher the EDL capacitance will be at negative potentials, and vice versa [27]. These investigations help improve our understanding towards the correlation between the nature of RTILs and their EDLs.

A vast majority of studies have focused on RTILs consisting of planar imidazolium-based cations and spherical (e.g., [BF_4_]^−^) or elliptical (e.g., [Tf_2_N]^−^) anions. However, it is still intriguing how the EDL structure and C-V curve will be modified when replacing anions with planar aromatic ones. In addition, the commonly adopted supercapacitor model in simulations consisting of two parallel electrodes with the in-between space filled by RTIL electrolytes, requires tuning the number of ion pairs in the channel repetitiously until the density fits the bulk properties; in contrast, one-electrode supercapacitor model, comprised of one charged electrode and RTIL electrolytes, with one end in contact with electrode surfaces and the other end of RTILs in contact with the vacuum, is facile to establish for molecular simulations and generally costs fewer computation resource. Thus, in this work, we used two different supercapacitor models including single-electrode model and two-electrode model (i.e., channel system) in simulations to compare the RTILs behaviors in EDLs. The main goal of this work is to investigate how the simulation model and anion shape could influence the microstructure and capacitance of the EDLs at the RTIL-electrode interfaces. The rest of this paper is organized as follows: the results on the EDL structure and capacitance are given in Section 2; the simulation models and methodology are presented in Section 3; finally, Section 4 summarizes this study.

## 2. Results

### 2.1. Which Supercapacitor Model Is Better?

**Figure 1:**
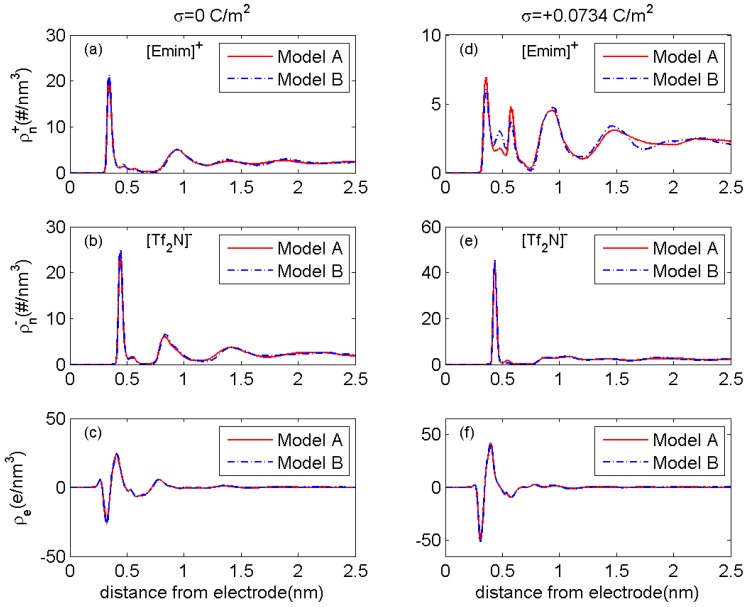
Ion number density and charge density profiles at 350 K. Number density of [Emim]^+^ near neutral (**a**) and electrified (**d**), electrodes with surface charge density σ = 0.0734 C/m^2^). Number density of [Tf_2_N]^−^ near neutral (**b**) and electrified (**e**) electrodes. Space charge density near neutral (**c**) and electrified (**f**) electrodes. The location of the cations and anions were computed on the basis of the geometrical center of the imidazolium ring and the entire anion, respectively.

**Scheme 1:**
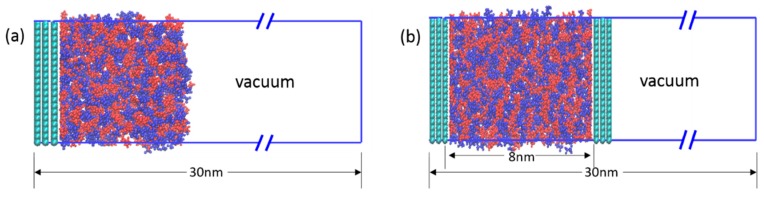

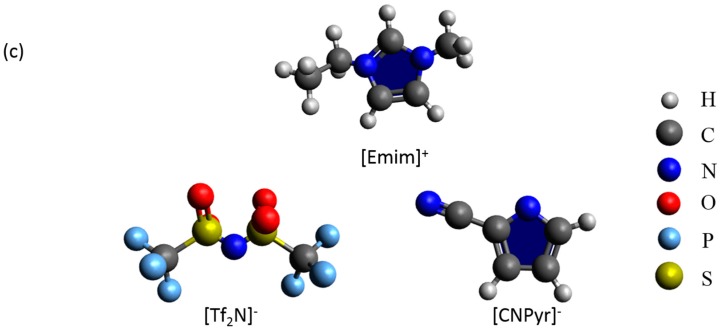
Snapshots of the supercapacitor models consisting of single electrode (Model A) in (**a**) and two opposite electrodes (Model B) in (**b**) in MD simulations. The box is 4.26 nm × 4.92 nm × 30 nm for both systems. The red and blue molecules in (**a**,**b**) represent cations and anions, respectively. The bottom panel (**c**) represents the molecular structures of ions constituting the ILs studied in this work. The cation is [Emim]^+^ with a five-membered ring, the anions are [Tf_2_N]^−^ which is elliptical (left) and [CNPyr]^−^ is an also planar five-membered ring (right).

Both one-electrode (Model A) and two-electrode (Model B) models as shown in Scheme 1 were employed, respectively, for simulating RTIL behaviors in EDLs near carbon electrode surfaces. Figure 1 shows the number density of [Emim]^+^ and [Tf_2_N]^−^ near the neutral and electrified electrodes of the two supercapacitor models. It is clear that both cations and anions are packed similarly in EDLs near electrode surfaces regardless of the supercapacitor model adopted. For example, the number density profiles of cations present two peaks in both systems near neutral electrode. The first peak of their density profile is located at ~0.350 nm from the electrode surface, which is almost the same as our previous work [26]. The number density values of the first peak for Model A (19.8 #/nm^3^) and B (20.2#/nm^3^) are very close to each other. Moreover, similar tendency is observed in their space charge density near electrodes (shown in Figure 1c,f) as well. Thereby, almost identical potential of zero charge (PZC) of Model A (−0.27 V) and B (−0.26 V) was obtained, respectively. It is reasonable to claim that the one-electrode supercapacitor model (Model A) exhibits almost identical EDL structure to the two-electrode model (Model B). In both systems, the ion layering of EDLs penetrates approximately 2~2.5 nm into the RTIL bulk region as report earlier [28]. It is also manifested that the influence of the two models on the EDL structure is actually dominated by interfacial properties of RTILs near electrodes, which is restricted to the electrode-electrolyte region of several nanometers in thickness. Thus, when the RTIL region of the supercapacitor models is sufficiently long in the normal direction of the electrode surface to contain both interfacial and bulk regions of RTILs, similar EDL structure properties can be obtained from both one-electrode and two-electrode models. Therefore, considering the facile model building and less expensive computation of simulating one-electrode system, Model A was employed in the following part of this work, since it could avoid the tedious work with high-time consumption on tuning the number of ion pairs in Model B [29,30,31].

### 2.2. Effects of Anion Shape on the EDL Microstructure

**Figure 2:**
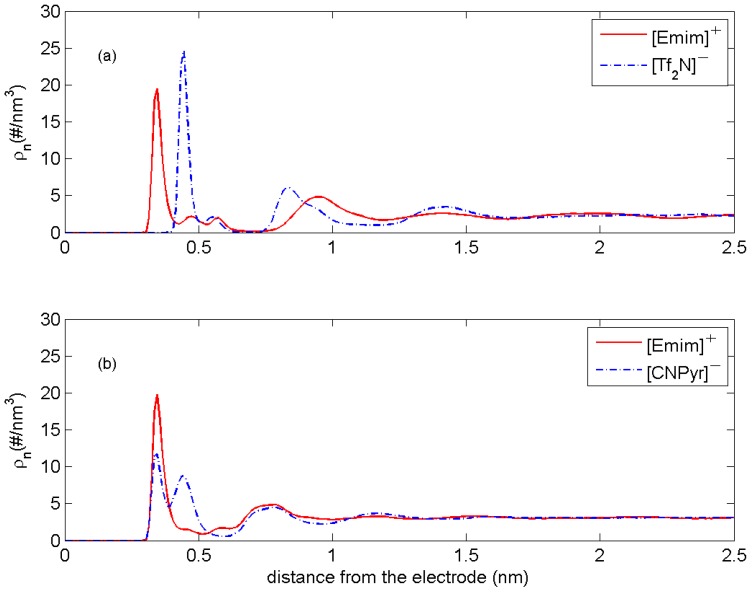
Ion number density profiles of [Emim][Tf_2_N] (**a**) and [Emim][CNPyr] (**b**) near neutral electrodes at 350 K.

**Figure 3:**
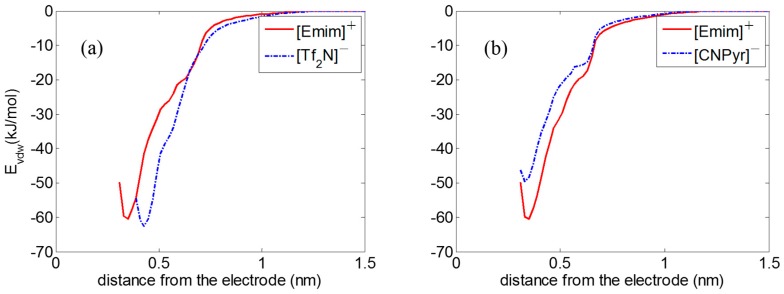
The van der Waals interaction energy between cations/anions and neutral graphene electrode for [Emim][Tf_2_N] and [Emim][CNPyr], respectively, at 350 K.

The EDL structures of [Emim][Tf_2_N] and [Emim][CNPyr] consisting of identical [Emim]^+^ cations and anions of different shapes were obtained using one-electrode supercapacitor models by MD simulations. Figure 2 shows the ion number density distributions of [Emim][Tf_2_N] and [Emim][CNPyr] near neutral electrode. There are several noticeable features in this figure. Two peaks of [Emim]^+^ are observed and the first peak is located at ~0.350 nm for both ILs. Their second peak is slightly different, i.e., the second peak of cations in [Emim][Tf_2_N] is further away from the electrode in contrast to that of [Emim][CNPyr]. Comparing the number density profiles of [Tf_2_N]^−^ and [CNPyr]^−^, there is only a single peak located at ~0.430 nm observed for [Tf_2_N]^−^, while twin peaks of [CNPyr]^−^ with the first peak located at ~0.330 nm are present, and the height of the first peak is lower than that of [Emim][Tf_2_N]. Such observations can be attributed to the strong interaction between planar [CNPyr]^−^ and graphene electrodes, especially the dominant van der Waals force between the electrode and ions [26]. To validate this, we calculated the van der Waals interaction potentials of ions and the graphene electrode, as shown in Figure 3.

Due to the strong π-π interaction between the planar [Emim]^+^ and the graphene wall [32,33], [Emim]^+^ ions were packed closer than [Tf_2_N]^−^, as shown in Figure 3a. In both [Emim][Tf_2_N] and [Emim][CNPyr], the van der Waals energy between [Emim]^+^ of the first ion layer at 0.350 nm and graphene is −62.6 kJ/mol. The strongest van der Waals interaction energy between the [Tf_2_N]^−^ and graphene electrode is located at *z* = 0.430 nm, accounting for the first ion layer of [Tf_2_N]^−^ packed at ~0.430 nm. Regarding [Emim][CNPyr], the van der Waals interaction energy depth between [CNPyr]^−^ and graphene is slightly lower (−49.7 kJ/mol) and [CNPyr]^−^ is closer (0.330 nm) towards graphene compared with that between [Emim]^+^ and graphene, which can be explained by the missing methyl and ethyl groups in planar [CNPyr]^−^ in comparison with [Emim]^+^, leading to more efficient packing of ions in the first layer. The second layer peaks of [CNPyr]^−^ can be resulted from the densely-packed cations in the first layer adjacent to the electrode, facilitating the formation of the second [CNPyr]^−^ layer to compensate the positive charges of the first cation layer and preventing the formation of second [Emim]^+^ layer [26].

**Figure 4:**
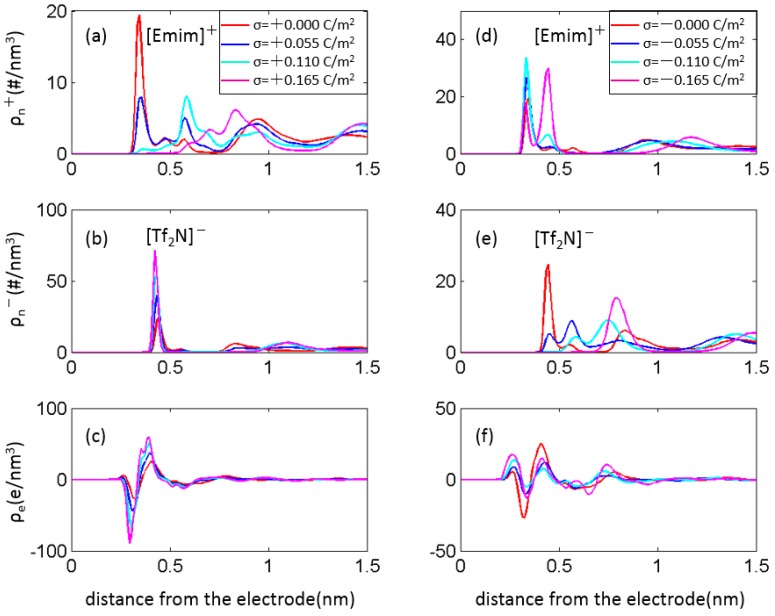
The number density distribution of cations and anions, and space charge density profiles of [Emim][Tf_2_N] near positively-charged electrodes (**a**–**c**) and negatively-charged electrodes (**d**–**f**).

**Figure 5:**
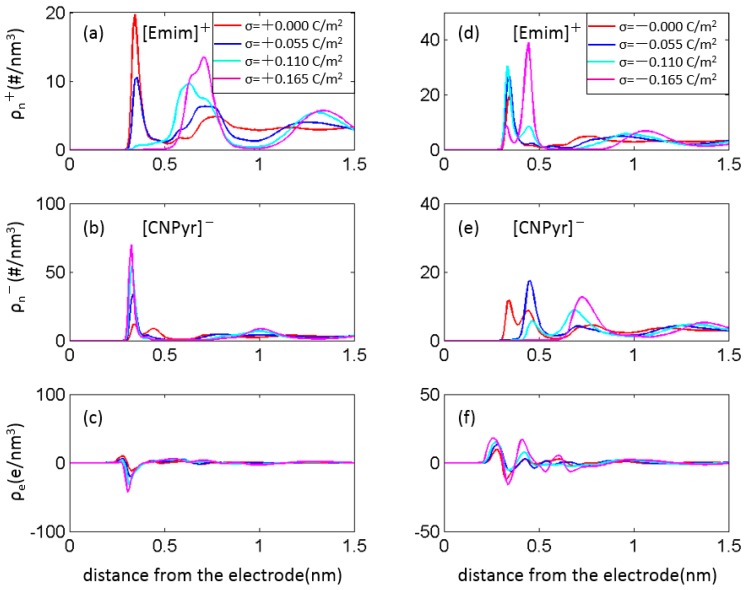
The number density distribution of cations and anions, and space charge density profiles of [Emim][CNPyr] near positively-charged electrodes (**a**–**c**) and negatively-charged electrodes (**d**–**f**).

Figure 4 and Figure 5 show the density distribution of cations and anions of [Emim][Tf_2_N] and [Emim][CNPyr] near neutral and charged electrodes, respectively. The cations in both [Emim][Tf_2_N] and [Emim][CNPyr] exhibit similar behavior at electrode surface regardless of the charge status of electrode. There is a significant accumulation of [Emim]^+^ ions at neutral electrode within the first counter-ion layer, which accounts for the strong affinity of the imidazolium ion toward the π-electronic graphene electrode [24,34,35,36]. With the increase of the negative charge density, the number of cations in the first layer is greatly increased until the saturation and leads to the occurrence of two cation layers near electrode. For instance, at σ = −0.110 C/m^2^, the density profile of [Emim]^+^ ions presents two peaks at *z* = 0.320 nm and *z* = 0.440 nm from the planar electrodes, similar to our previous work [26]. It is also noticed that the distance between the two peaks is 0.120 nm, which is approximately one half of the diameter of the imidazolium ring, suggesting that the planar rings change from parallel to perpendicular alignment near the electrode. The transition of the cation orientation is shown below. Nevertheless, anions of [Emim][Tf_2_N] and [Emim][CNPyr] exhibit dissimilar behaviors at electrode surfaces. [CNPyr]^−^ anions are located closer towards electrode surfaces than [Tf_2_N]^−^ at varying charge densities, which could be attributed to the specific orientation of [CNPyr]^−^ in EDLs as illustrated below.

**Figure 6:**
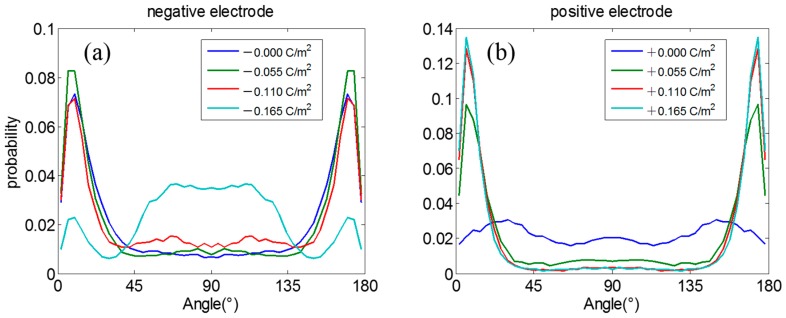
(**a**) The orientation distribution probability of the angle formed between the cation [Emim]^+^ and the negatively-charged electrode; (**b**) the orientation distribution probability of the angle formed between the anion [CNPyr]^−^ and the positively-charged electrode at varying charge densities.

To elucidate the arrangement of ions in RTIL-based EDLs, the ion orientation, defined by the angle formed by the normal vector of the plane of [Emim]^+^ cations or [CNPyr]^−^ anions and the normal vector of the electrode, was computed as shown in Figure 6. At lower surface charge density, the [Emim]^+^ cation is parallel to the graphene electrode, while at higher surface charge density, the orientation of [Emim]^+^ changes from parallel to random alignment. We attribute this transition to that lessening the overlapping of the π orbital of the imidazolium cation with the vacant orbital of the graphene electrode leads to more efficient packing of cations to compensate the negative charges [23,24]. On the contrary to [Emim]^+^, [CNPyr]^−^ anions are more parallel to the positive electrode as the electrode is more positively charged, which can be explained by the reduced steric effects resulting from the absence of methyl and ethyl groups in [CNPyr]^−^ in contrast to [Emim]^+^.

### 2.3. Effects of Anion Shape on EDL Capacitance

**Figure 7:**
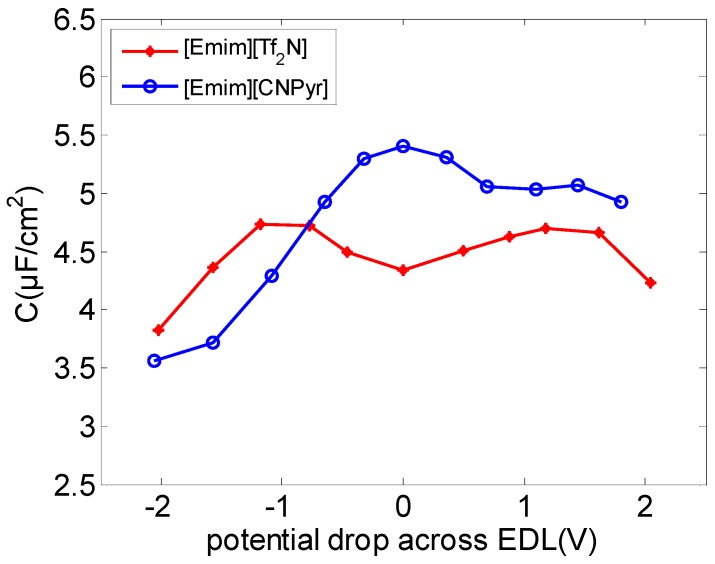
The differential capacitance versus the potential drop (V) across the EDLs of [Emim][Tf_2_N] and [Emim][CNPyr], respectively, at 350 K.

To better understand the influence of the anion shape of RTIL electrolytes on the EDL capacitance, the differential capacitance (C) vs. electrical potential drop (V) across EDL was shown in Figure 7. It is obvious that the C-V curves of [Emim][Tf_2_N] and [Emim][CNPyr] are quite different and [Emim][Tf_2_N] exhibits a camel-like shaped C-V curve, whereas [Emim][CNPyr] displays an incomplete camel-shaped C-V curve. Similarly, Smith’s group [37] also reported camel-shaped C-V curve of [Emim][Tf_2_N] electrolytes-based supercapacitors using flat graphene electrode by MD simulations, consistent with the experimental measurement with similar cations by Lockett et al. [24,38]. The parallel orientation of [CNPyr]^−^, resulted from the π-π interaction with graphene, leads to more effective screening by the countercharges on electrode surfaces. Thereby, the anion-dominated capacitance at positive potentials for [Emim][CNPyr] is higher than that for [Emim][Tf_2_N]. However, at highly negative potentials, anions are gradually squeezed out from the EDL by cations, and more cations of [Emim][Tf_2_N] accumulated to replace anions than those of [Emim][CNPyr] in EDLs due to the relative larger space occupied by [Tf_2_N]^−^, leading to the higher capacitance of [Emim][Tf_2_N] at negative potentials than [Emim][CNPyr].

**Figure 8:**
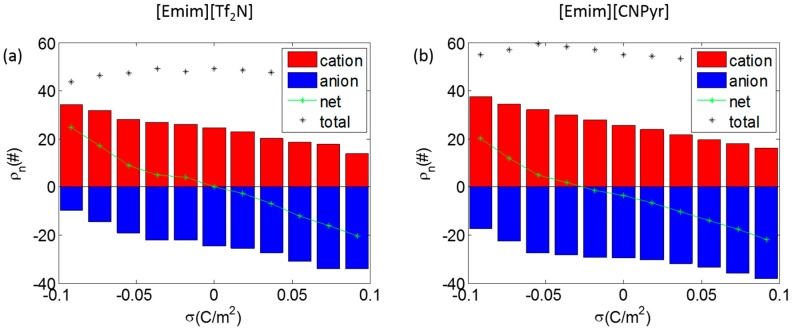
The number of ions within the first layer of EDLs for the [Emim][Tf_2_N] (**a**) and [Emim][CNPyr] (**b**) near positively-charged electrodes and negatively-charged electrodes at 350 K.

The number of accumulated co-ions and counter-ions in EDLs of [Emim][Tf_2_N] and [Emim][CNPyr] of the first layer at varying charge densities can also explain the origin of the shape of C-V curves. According to Figure 8, it is apparent that there are more cations and anions accumulated into EDLs of [Emim][CNPyr] than those of [Emim][Tf_2_N] at different charge densities, indicating the more efficient packing of ions with planar geometry in EDLs, resulting from the π-π interaction. Moreover, as the surface charge density increases, the number of counter-ions is increased while the number of co-ions is decreased in EDLs for both RTILs, consistent with previous observations [39,40]. Nevertheless, the negative net charges in EDLs of [Emim][CNPyr] are also higher than those of [Emim][Tf_2_N] at fixed positive surface charge densities of the electrodes. In addition, the variations in negative net charges of EDLs per unit charge density for [Emim][CNPyr] are steeper than [Emim][Tf_2_N], leading to the higher capacitance at positive potentials of the C-V curve. Furthermore, the positive net charges of [Emim][CNPyr] are lower than those of [Emim][Tf_2_N] at higher surface charge densities, resulting in the smaller capacitance.

## 3. Materials and Methods

Two supercapacitor models, including one-electrode model (Model A) and two-electrode model (Model B) utilized in MD simulations, were shown in Scheme 1. The simulation box is 4.26 nm, 4.92 nm, and 30 nm in *x*-, *y*-, and *z*-directions, respectively. In Model A, ILs were initially placed on the graphene surface and the graphene surface. In Model B, two electrodes were separated by an 8 nm distance with the gap filled by RTILs, and the rest of the simulation box is a 22 nm slab of vacuum. The graphene surface is parallel to the xy-plane and kept frozen in all simulations. A schematic representation of cations and anions simulated in this work is shown in Scheme 1c, where the cation 1-ethyl-3-methylimidazolium ([Emim]^+^) with a planar ring and two alkyl groups, the anion 2-(cyano)pyrrolide ([CNPyr]^−^) composed of a planar ring, and bis(trifluoromethanesulfonyl)imide ([Tf_2_N]^−^) anion were included. The force fields for all ions were taken from [41]. Particularly, the total partial charges of the cation and the anion are ±0.8 e, respectively.

All of our simulations were performed in NVT ensemble with the MD package Gromacs 4.6 [42]. The temperature was maintained by using a Nosé-Hoover coupling [43] with a time constant of 0.5 ps. For Model A, 300 pairs of RTILs were included, while the number of the ion pairs in Model B was adjusted until the density in the channel center matched that of the bulk RTIL with a deviation of 0.1%. The coulombic interactions were computed using slab-PME to ensure the accuracy of the electrostatic force [44]. To compute the electrostatic interactions in reciprocal space, an FFT grid spacing of 0.12 nm and cubic interpolation were used. A coulombic cutoff of 1.2 nm was adopted to calculate the electrostatic interactions in the real space. The non-electrostatic interactions were computed by direct summation with a cutoff length of 1.2 nm. For each simulation, the system was initialized at 800 K for 1 ns, followed by annealing and equilibration of 30 ns at 350 K. The last 10 ns trajectories were used for further analysis.

The electrical potential distribution was calculated using the Poisson equation:

(1) $$\frac{d^{2}\varphi }{dz^{2}}=-\frac{\rho_{e}\left(z\right)}{\varepsilon_{0}}$$

where *ρ*_e_(*z*) and ε_0_ are the space charge density and the vacuum permittivity, respectively. By integrating Equation (1) twice, the electrical potential distribution can be obtained using:

(2) $$\varphi \left(z\right)=\frac{\sigma }{\varepsilon_{0}}-\frac{1}{\varepsilon_{0}}\int_{0}^{z}\left(z-z^{\prime }\right)\rho_{e}\left(z^{\prime }\right)dz^{\prime }$$

where *σ* is the surface charge density. Therefore, the potential drop across the EDL is calculated as:

(3) $$\varphi_{EDL}=\varphi -\varphi_{PZC}$$

In this work, the differential capacitance is calculated by using C = d*σ*/dV_EDL_. Here, the σ-V_EDL_ correlation was first fitted to a fourth-order polynomial and the differential capacitance was then obtained by analytical differentiation of the polynomial.

## 4. Conclusions

In this work, MD simulations were performed to study the effects of different supercapacitor models and anion shapes on the EDL microstructure and capacitance of RTIL’s electrolyte. It was revealed that either the single-electrode supercapacitor model or the two-electrode model gave rise to nearly identical EDL structure. Thus, single-electrode model with low computational cost was adopted to investigate the effects of [Tf_2_N]^−^ and planar [CNPyr]^−^ anions on the capacitive performance of [Emim][Tf_2_N]- and [Emim][CNPyr]-based supercapacitors. The results demonstrate that [Emim]^+^ cations in EDLs of both RTILs exhibit similar behaviors. Nevertheless, there are obvious differences in the interfacial properties of [Tf_2_N]^−^ and [CNPyr]^−^ anions across EDLs. [CNPyr]^−^ anions are located closer to electrode surfaces in contrast to [Tf_2_N]^−^ due to the π-π interactions with graphene. It was further verified that [CNPyr]^−^ anions prefer to align parallel to graphene with the increase of electrode surface charge density, similar to planar [Emim]^+^ cations at moderate potential. Accordingly, different C-V curves of [Emim][Tf_2_N] and [Emim][CNPyr] are present, especially at positive potentials. The more efficient packing of [CNPyr]^−^ anions within the EDLs results in higher capacitance of [Emim][CNPyr] than [Emim][Tf_2_N] at positively-charged electrodes. The corresponding number of ions accumulated in EDLs also evidenced the origination of the shape of the C-V curves of two RTILs. It is noteworthy that the planar ion geometry plays a similar role to the small-sized ions [24,26] in terms of facilitating the ion packing in EDLs and, thus, enhancing the capacitance. Additionally, experimental evidence is required in future work to validate the findings from our MD simulation. However, it is challenging for experiments to use the perfect planar graphene electrode in supercapacitors. Most of experiments adopt porous carbon or glassy carbon electrodes for measurement, whose performance has also been successfully predicted or explained by MD simulations [1]. According to Smith et al. [37], the simulation results in principle should be able to validate or predict the experimental measurements, assuming that the equivalent system is investigated in experiments and simulations. Therefore, even though all of the findings in this work are established on theoretical computations, it is reasonable to believe that our simulations, which have successfully predicted the capacitive performance of imidazolium bis(trifluoromethylsulfonyl)imide electrolytes-based supercapacitors [24,37,38] can provide reliable prediction for [Emim][CNPyr], as well.
